# Therapeutic Innovation from Plant-Derived Thai Herbal Extracts: α-Glucosidase Inhibitory Activity, Mechanistic Insights and Formulation Potential of the Selected Thai Rejuvenation Remedy

**DOI:** 10.3390/life16071084

**Published:** 2026-06-28

**Authors:** Suthinee Sangkanu, Thanet Pitakbut, Chotika Buekhuntod, Sathianpong Phoopha, Jiraporn Khanansuk, Wandee Udomuksorn, Kasemsiri Chandarajoti, Sukanya Dej-adisai

**Affiliations:** 1 Department of Pharmacognosy and Pharmaceutical Botany, Faculty of Pharmaceutical Sciences, Prince of Songkla University, Hat Yai, Songkhla 90112, Thailand; suthinee.s@psu.ac.th (S.S.); chotika.lp@hotmail.com (C.B.); jiraporn.kha@psu.ac.th (J.K.); 2 Pharmaceutical Biology, Department of Biology, Friedrich-Alexander-Universität Erlangen-Nürnberg (FAU), 91058 Erlangen, Germany; thanet.pitakbut@fau.de; 3 Department of Evolution and Population Biology, Institute for Biodiversity and Ecosystem Dynamics, University of Amsterdam, 1098 XH Amsterdam, The Netherlands; 4 Department of Traditional Thai Medicine, Faculty of Health and Sports Science, Thaksin University, Phatthalung 93210, Thailand; 5 Traditional Thai Medical Research and Innovation Center, Faculty of Traditional Thai Medicine, Prince of Songkla University, Hat Yai, Songkhla 90112, Thailand; sathianpong.p@psu.ac.th; 6 Division of Health and Applied Science, Faculty of Science, Prince of Songkla University, Hat-Yai, Songkhla 90112, Thailand; wandee.u@psu.ac.th; 7 Department of Pharmaceutical Chemistry, Faculty of Pharmaceutical Sciences, Prince of Songkla University, Hat Yai, Songkhla 90112, Thailand; kasemsiri.c@psu.ac.th

**Keywords:** Thai Rejuvenation Remedies, α-glucosidase inhibitor, antidiabetic, phytochemical analysis, molecular docking, density functional theory (DFT), piperine, cyclodextrin

## Abstract

This study highlights the therapeutic innovation potential of bioactive plant extracts derived from the selected Thai Rejuvenation Remedy 2 (TRJ 2) for antidiabetic applications. By integrating phytochemical profiling with in vitro α-glucosidase inhibition assays and in silico analyses, including molecular docking and density functional theory (DFT), a comprehensive evaluation of the extract’s bioactivity and mechanistic basis was achieved. The findings demonstrate that both the relative abundance and chemical reactivity of constituent compounds contribute to the overall inhibitory effect through synergistic and competitive interactions. Piperine was identified as a potential bioactive metabolite, with a theoretically strong binding affinity and high reactivity toward the target enzyme. Among the tested extracts, the 80% ethanol extract exhibited the highest inhibitory activity (IC_50_ = 34.32 µg/mL), underscoring the importance of extraction optimization for maximizing therapeutic efficacy. Furthermore, formulation of the extract with cyclodextrin significantly enhanced solubility and improved pharmaceutical characteristics, meeting the quality requirements of the Thai Herbal Pharmacopoeia (THP). However, the detection of cadmium slightly above permissible levels indicates the need for stricter raw material quality control. Overall, TRJ 2 represents a promising source of bioactive plant-derived compounds and a viable candidate for development into innovative, ready-to-use herbal therapeutics for diabetes management.

## 1. Introduction

The rapidly increasing number of elderly people worldwide has resulted in significant compromises, particularly an increase in the prevalence of age-related diseases. Noncommunicable diseases (NCDs) such as diabetes, musculoskeletal disorders, cardiovascular and neurological conditions, and cancers increase with age and place substantial burdens on individuals and healthcare systems [1]. In 2021, a study examined five behavioral risk factors for noncommunicable diseases in Thailand. The most common risk was an unhealthy diet (56.93%), followed by overweight or obesity (50.03%), physical inactivity (42.70%), alcohol consumption (29.73%), and smoking (16.61%) [2]. Obesity and overweight are significant risk factors for diabetes, particularly type 2 diabetes (T2DM). Globally, the prevalence of diabetes that occurs in adults has risen from 4.3% to 9.0% for males and from 5.0% to 7.9% for women. While both type 1 diabetes mellitus (T1DM) and T2DM are contributing to the rising prevalence, T2DM is thought to be causing 85% to 95% of the increase [3]. Early diagnosis is crucial to avoid T2DM and its short-term and long-term metabolic and cardiovascular. Additionally, the risk of developing T2DM can be decreased in people with pre-diabetes by managing their condition with dietary and lifestyle changes, as well as, when necessary, pharmaceutical treatment [4].

T2DM is characterized by elevated insulin levels, insulin resistance, and progressive β-cell dysfunction. Its development involves multiple organs, including the pancreas (β- and α-cells), liver, skeletal muscle, kidneys, brain, small intestine, and adipose tissue [5]. The diagnosis can be made using any of the following criteria: a random plasma glucose of ≥200 mg/dL with classic hyperglycemic symptoms, a fasting plasma glucose of ≥126 mg/dL after an 8 h fast, a 2 h plasma glucose of ≥200 mg/dL following a 75 g oral glucose load, or an hemoglobin A1c (HbA1c) of ≥6.5% [6]. Pre-diabetes is a disease with elevated blood sugar levels but not high enough to be categorized as diabetes. Pre-diabetic criterion is fasting plasma glucose between 100 and 125 mg/dL after an 8 h fast or a 2 h plasma glucose between 140 and 199 mg/dL a 75 g oral glucose load, or HbA1c of 5.7% to 6.4% [7]. However, individuals with unmanaged pre-diabetes may eventually acquire T2DM. The approach is most likely to be effective for decreasing the percentage of prediabetic patients who develop T2DM during a four-year period from 33% to 20% is a combination of dietary and physical activity improvements [8].

The traditional medicine system of plant extracts has developed and widely utilized for the management of various health conditions. It is estimated that approximately 80-85% of the population relies on plant-derived extracts or their active constituents as a form of traditional healthcare to address primary medical needs. Several medicinal plants are employed in conjunction with conventional pharmacotherapy to treat chronic illnesses, including diabetes mellitus. For example, plants such as *Coccinia indica*, *Momordica charantia*, and *Cinnamomum verum* have demonstrated glucose-lowering effects in both in vitro and in vivo studies [9,10]. Reportedly, patients with T2DM tend to use herbal medicine because of concerns about the possible side effects of antidiabetic drug and their personal belief that herbal remedies from natural sources are safer and more effective. However, healthcare providers have often demonstrated minimal acceptance of herbal medicine to manage diabetes. This could be attributed to the insufficiency of well-designed scientific reports or long-term clinical trial studies on efficiency and possible adverse side effects [11]. Although many medicinal plants used in traditional medicine are well known to possess glucose-lowering properties, previous studies have largely focused on single plant extracts. There remains insufficient evidence regarding the synergistic interactions among phytochemical constituents in complex traditional formulations, the molecular mechanisms underlying α-glucosidase inhibition, and the translation of herbal extracts into stable, standardized, and pharmaceutically acceptable dosage forms. Furthermore, previous studies rarely combined computational chemistry, biological tests, and phytochemical profiling into an integrated framework to explain both efficacy and mechanism of action.

In Thailand, the traditional remedy known as the “Elixir of Life” has long been utilized as a rejuvenating medicine, with its formulations preserved through historical documentation and traditional knowledge. Therefore, the present study aims to conduct an in-depth investigation of the bioactive constituents present in effective antidiabetic herbal formulations and to develop these extracts into appropriate pharmaceutical preparations. This objective is particularly important because many bioactive phytochemicals exhibit poor aqueous solubility and limited absorption, thereby restricting their practical therapeutic applicability and clinical effectiveness. This study, we examined 62 traditional medicine formulations to evaluate their potential to inhibit the activity of the enzyme α-glucosidase. We found that the Thai Rejuvenating Remedy 2 (TRJ 2) showed promising anti-enzyme activity. Therefore, this research selected TRJ 2 to analyze its chemical components, predict the activity of compounds by molecular docking and develop it into a modern medicinal form suitable for easy consumption. Additionally, the study included the evaluation of biomarker substances to determine the quality of the recipe.

## 2. Materials and Methods

### 2.1. Thai Rejuvenating Remedy Extraction

The dried herbs (10 kg of *Piper sarmentosum* Roxb. (root), 10 kg of *Piper ribesioides* Wall. (climber), and 10 kg of *Zingiber officinale* Rose. (rhizome)) from Thai rejuvenating remedy 2 (TRJ 2) were purchased from a local herbal shop in Songkhla province, Thailand. The single herb (1 kg) was submerged in 80% ethanol and maintained at room temperature for 72 h, with thorough stirring every 24 h. After this period, the solution was filtered through filter paper to collect the extract. The remaining plant material was re-submerged in fresh solvent, and this process was repeated three times. The combined filtrates were evaporated using a rotary evaporator (Hei-Vap Value, Heidolph, Schwabach, Germany) at 55 °C and then dried further in a water bath at 55 °C.

Thai Rejuvenating Remedy 2 (TRJ 2) (12 kg) was prepared by combining each constituent herb in equal proportions (1:1:1 ratio), followed by extraction utilizing the same methodology employed for individual herbs. The resulting extract was weighed to determine the extraction yield as a percentage of the initial herb weight. Additionally, TRJ 2 was subjected to extraction using various solvents such as hexane, ethyl acetate, ethanol, and water to facilitate a comparative evaluation of its anti-α-glucosidase and associated biomarkers. The extraction protocol started with maceration of TRJ 2 (12 kg) in hexane, followed by a procedure that employed within 80% ethanol. After the hexane extraction, the residual material was further subjected to extraction with ethyl acetate, ethanol and water. The resulting extracts were then stored at 4 °C for future biological and analytical studies.

### 2.2. α-Glucosidase Inhibition

Fifty microliters of single plant extracts and TRJ 2 extracts at a concentration of 2 mg/mL were incubated with 50 μL of α-glucosidase enzyme (1 unit from *Saccharomyces cerevisiae*, Type I, lyophilized powder, Sigma, EC 3.2.1.20, St. Louis, MO, USA) for 2 min at 37 °C. This mixture was combined with an additional 50 μL of 0.1 M phosphate buffer (pH 7.0). The reaction was initiated by adding 50 μL of *p*-nitrophenyl-α-D-glucopyranoside (*p*NPG) as the substrate. The formation of *p*-nitrophenol (*p*NP) was monitored at 405 nm every 30 s over a 10 min period using a microplate reader (VARI-OSKAN LUX, Thermo Scientific, Hudson, NH, USA). The absorbance readings of the test wells were corrected by subtracting the absorbance of the blank prior to calculating the reaction velocity (Equation (1)). The initial reaction velocity (V) for each sample was determined, and the percentage inhibition was calculated with Equation 2. The IC_50_ value of extract and standard piperine were derived from a calibration curve plotting percentage inhibition against seven different sample concentrations (ranging from 15.62 to 1000 μg/mL). All experiments were conducted in triplicate [12].

Velocity = ∆ Absorbance at 405 nm/∆ Time (1)

% Inhibition = [(Vcontrol − Vsample)/Vcontrol]/100 (2)

### 2.3. Gas Chromatography-Mass Spectrometry (GC–MS) Analysis

Gas chromatography-mass spectrometry (GC–MS) analysis of extracts from TRJ 2 was conducted using an Agilent Technologies 7890 B GC system coupled with a 5977B Mass Selective Detector (MS) (Agilent Technologies, Waldbronn, Germany). Separation was performed on a VF WAXms capillary column (30 m × 250 × 0.25 μm) with helium as the carrier gas at a flow rate of 1 mL/min. The column temperature program started at 60 °C, then increased to 160 °C at a rate of 10 °C/min, followed by a rise to 250 °C at 2.5 °C/min, with a 15 min hold at the final temperature. The MS operated in electron ionization mode at 70 eV, with the ion source temperature set at 230 °C, scanning continuously from 35 to 500 *m*/*z*. The chemical compounds were identified by matching their mass spectra to those in the Wiley and NIST14 spectral libraries.

### 2.4. Molecular Docking Simulation Methodology

In this study, molecular docking was performed using our previously published protocol [13,14]. In brief, we used AutoDock Vina version 1.2.5 [15] to perform docking simulations. First, the crystal structure of α-glucosidase (PDB ID: 3A4A, [16]) was downloaded from the PDB. Water, ions, and non-relevant molecules were removed using the USFC Chimera program (version 1.17.3) [17]. Then, the α-glucosidase protein structure was separated from its co-crystallized ligand (glucose molecules). These separated molecules were used for re-docking validation to ensure the reliability of the established docking protocol. In this step, the researchers used the AutoDock Tool version 1.5.6 [18] to prepare proper input files for both the enzyme and the extracted native glucose molecule. Meanwhile, the 2D chemical structures of all compounds of interest were downloaded from the PubChem database, and Open Babel version 3.1.0 [19] was used to generate optimized 3D structures using the General Amber Force Field (GAFF) and to prepare suitable input files for the docking simulations.

In the experimental setup, the authors followed our previous reports [13,14] and defined the alpha-glucosidase catalytic pocket as the docking site. The coordinates and dimensions of the defined site were set to 21.2, −7.5, and 24.3, and 18 Å × 18 Å × 18 Å. The authors used default values in nearly all docking parameters, except for exhaustiveness and the number of models. The exhaustiveness was set to 10, and the number of models to 20. Before applying this protocol, it was validated using the redocking method, in which the extracted co-crystallized ligand was redocked to its original position within the protein structure to ensure the docking experiment setup had strong predictive power and a high correlation with a biological experiment. The current docking protocol met the standard acceptance criterion, with the root-mean-square deviation (RMSD) between the redocked and original ligand poses being less than 3 Å [13,14].

For post-docking analysis, only one promising docking pose was selected for each compound. The selection criterion was based on the assumption that compounds with similar chemophores interact with the target protein in a similar manner.

### 2.5. Density Function Theory (DFT) Calculation

The authors used ORCA version 6.0.0 [20] to calculate molecular DFT descriptors, including the highest occupied molecular orbital (HOMO) and the lowest unoccupied molecular orbital (LUMO) energy levels, the HOMO-LUMO energy gap, and molecular hardness. The two-step single-point energy and property calculation approach was used. First, the authors performed 3D molecular-geometrical optimization using the B3LYP-D4/def2-SVP method for each compound of interest [21]. Second, the atom coordinates of each geometrically optimized structure were used as input for the second step. In this second step, the authors used the PBE0-D4/def2-TZVP method [22] with RIJCOSX and TightSCF functions to calculate single-point energy and properties. Finally, the results were visualized using the Avogadro program version 1.2.0 [23].

### 2.6. Linear Regression Relationship Between Anti- α-Glucosidase Activities and the Remaining Predicted Strong Inhibitor Content in the TRJ 2 Remedy

The linear regression analysis was conducted in MS Excel as prescribed in the authors’ previous article [24]. Both % inhibition and IC_50_ values were plotted on the Y-axis of the separate graph to represent the anti-α-glucosidase activity obtained in this study. The remaining content of the TRJ 2 predicted strong inhibitors was calculated using Equation (3) described below and plotted on the X-axis.

Remaining content of the predicted strong inhibitors =  total predicted strong inhibitor content − total predicted weak inhibitor content,(3)

The determined linear regression relationship was presented in Equation (4)

y = mx + c,(4)

While y represents the obtained anti-α-glucosidase activity (either %inhibition or IC_50_ value), m is the regression coefficient (the rate of change in y relative to x), x equals the remaining content of the predicted strong inhibitors from Equation (3), and c represents the baseline value of y when x is absent. The R^2^ value was used to evaluate the linear equation and the relationship between anti-α-glucosidase activities and the remaining predicted strong-inhibitor content from the TRJ 2 remedy.

### 2.7. Preparation for TRJ 2 Complex

A complex powder was synthesized through the homogeneous blending of TRJ 2 with β-cyclodextrin, maltodextrin, acacia gum, and pectin at ratios of 1:2, 1:2, 1:4, and 1:4, respectively. The resulting mixture was subjected to vacuum drying (Vacuubrand PC 3/RZ 6, Wertheim, Germany) for 5 h or until complete dry was achieved, following thorough grinding. Then, the dried complex was ground into a fine powder and accurately weighed for subsequent analyses.

### 2.8. Quality Control of Formulated Capsule

#### 2.8.1. HPLC Analysis of Piperine in Extracts and TRJ 2 Capsules

The samples, each approximately 10 mg in weight, were combined with 1 mL of solvent and subsequently filtered through a 0.22 µm membrane paper. Analysis was performed using HPLC system included a Hitachi Chromaster with a pump (model 5110), UV/Vis detector (model 5410), oven (model 5310), and autosampler (5210 model) (Hitachi High-Tech Science, Tokyo, Japan). The separation was carried out on a Fortis C18 column (250 mm × 4.6 mm, 5 µm particle size). The mobile phase consisted of methanol and 0.1% acetic acid in water at 50:50 (gradient), pumped at a flow rate of 1.00 mL/min. A 10 µL aliquot of each sample was injected into the system at 40 °C. The analysis was limited to a maximum run time of 15 min, with detection occurring at 340 nm. A calibration curve was generated to quantify the amount of piperine in the samples. Piperine was identified by comparison of its retention time with that of the reference standard. Quantification was performed using an external standard calibration curve prepared from serial dilutions of piperine standard solutions. The calibration curve showed good linearity over the tested concentration range, with a correlation coefficient (R^2^) of 0.9454. The retention time of piperine was approximately 11.850 to 11.877 min. All samples were analyzed in triplicate, and the results were expressed as mean ± standard deviation.

#### 2.8.2. Weight Variation

A uniformity of weight test was conducted in accordance with the standards specified in the Thai Herbal Pharmacopoeia (THP) [25]. Ten capsules were randomly selected and individually weighed. Subsequently, each capsule was carefully opened without losing any part of the shell, and its contents were removed as completely as possible. The capsules were then cleaned using a small brush, and the weight of the capsule shells was recorded. The weight of the contents within each capsule was determined by calculating the difference between the total capsule weight and the shell weight. The weight of the contents in each capsule was compared with the labeled weight. The THP limit for weight variation in the case of capsule weighting is not more than two of the individual weights deviate from the labeled weight or the average weight by more than the percentage deviation of 10%, and none deviates by more than twice that percentage.

#### 2.8.3. Microbial Limit Tests

Microbial limit tests were performed following THP 2021 [25]. A 1 g sample of the powder was suspended in 9 mL of phosphate buffer solution at pH 7.2. Serial dilutions were prepared, and microbial viability was determined using the spread plate technique. The inoculated plates were incubated at 37 °C from 1 to 5 days. Microbial enumeration was obtained by averaging the results from triplicate determinations. The media employed for microbial analysis included Mueller-Hinton Agar (MHA) for Total Aerobic Microbial Count (TAMC), Sabouraud Dextrose Agar (SDA) for Total Yeasts and Molds Count (TYMC), and Eosin Methylene Blue (EMB) agar for the enumeration of Bile-tolerant Gram-negative bacteria. The detection of specific bacterial species, such as *Escherichia coli*, *Clostridium* spp., and *Salmonella* spp., was conducted using the spread plate method on EMB, Cooked Meat Medium (RCM), and *Salmonella*-*Shigella* (SS) agar, respectively.

#### 2.8.4. Analysis of Heavy Metals Contamination

Five grams of the samples were ground to a fine powder. The powdered samples were then dried at 55 °C for 6 to 8 h in an oven to eliminate moisture content. Immediately following the drying process, an accurately measured 3 g of the sample was transferred into a 250 mL beaker and subjected to digestion with 20 mL of 30% nitric acid on a hot plate at 60 °C for 30 min. Subsequently, 20 mL of 30% nitric acid was added, and the mixture was maintained for 3 h until a clear solution was obtained, with the final volume reduced to less than 10 mL. The digested solution was then transferred to a 10 mL volumetric flask and diluted to volume with deionized water. Standard solutions for arsenic (As), lead (Pb), cadmium (Cd), and mercury (Hg) were prepared through dilutions of stock solutions with a concentration of 1000 mg/mL. Then, the prepared samples and the standards were analyzed to determine the concentrations of the heavy metals using the atomic emission spectrometry (ContrAA 800-High-End AAS Spectrometer, Analytik Jena, Tewksbury, MA, USA) [26].

#### 2.8.5. In Vitro Dissolution Studies

In vitro dissolution studies for TRJ 2 (250 mg/capsule) were conducted utilizing a USP Type-II dissolution apparatus (EDT 08LX Electrolab, Mumbai, India). The dissolution procedures employed 900 mL of simulated gastric fluid (pH 1.3; containing 2 g NaCl, 2 M HCl, and deionized water up to 1000 mL) and intestinal buffer solution (pH 6.8; containing 88.05 g KH_2_PO_4_, 8.96 g NaOH, and deionized water up to 1000 mL). The experiments were performed at a controlled temperature of 37 ± 0.5 °C with the paddle rotation set at 100 rpm. At specified intervals of 15, 30, 45 and 60 min, aliquots of 10 mL were withdrawn from the dissolution medium to assess biomarker release [27]. Following each sampling, an equivalent volume of fresh buffer was added to maintain the volume consistency. The withdrawn samples were filtered through a 0.22 µm membrane filter prior to analysis by HPLC.

#### 2.8.6. Stability

Stability assessments of TRJ 2 complexes were conducted within a stability incubator (Yamato, Model IN604/804, Tokyo, Japan) by storing 10 capsules in screw-capped glass vials under controlled temperature and humidity conditions. The samples were tested to 6 cycle accelerated stability protocol consisting of 24 h at 4 °C and 24 h at 45 °C. The physical characteristics and biomarker composition of the TRJ 2 complexes in three capsules were assessed at the end of the program by HPLC.

## 3. Results

### 3.1. Extraction and Determination of α-Glucosidase Inhibition

The three herbal plants of TRJ 2 were extracted using 80% ethanol, *Z. officinale* produced the highest extraction percentage (13.57%), followed by *P. sarmentosum* (6.27%) and *P. ribesioides* (5.50%). 80% ethanol extraction produced the highest yield of TRJ 2 (11.59%), followed by ethanol, water, hexane, and ethyl acetate.

α-glucosidase inhibition was then evaluated at a concentration of 2 mg/mL. Except for TRJ 2, which was extracted using water, all TRJ 2 extracts showed an inhibitory rate of greater than 70%. Among the various single herbs, *P. ribesioides* (97.78% ± 0.76%) and *P. sarmentosum* (92.74% ± 0.69%) showed better inhibitory efficacy than *Z. officinale* (51.86% ± 0.87%). The IC_50_ values of extracts with *α*-glucosidase inhibition greater than 70% were determined by further analysis. The findings indicated that *P. ribesioides* and TRJ 2 extraction with 80% ethanol possessed the lowest IC_50_ values at 21.99 µg/mL and 34.32 µg/mL, respectively, in comparison to the positive control, acarbose, which had an IC_50_ of 242.94 µg/mL (Table 1).

### 3.2. Gas Chromatography-Mass Spectroscopic Analysis of TRJ 2 and Herbs

The chemical composition of TRJ 2 extracts obtained using various solvents was analyzed by gas chromatography-mass spectrometry (GC-MS) (Table 2). The analysis revealed three compounds consistently present in all extracts: (E)-1-(4-hydroxy-3-methoxyphenyl)dec-3-en-5-one ([6]-isoshogaol), 1-(4-hydroxy-3-methoxyphenyl)dec-4-en-3-one ([6]-shogaol), and 5-hydroxy-1-(4-hydroxy-3-methoxyphenyl)decan-3-one ([6]-gingerol). Among these, [6]-shogaol was identified as the predominant compound in TRJ 2 extracts prepared with 80% ethanol, hexane, ethyl acetate, and ethanol. In contrast, the extract obtained with water showed 1,2-cyclopentanedione as the most abundant component.

Considering the chemical profile of TRJ 2 extract obtained with 80% ethanol, which demonstrated the most potent α-glucosidase inhibitory activity (IC50 = 34.32 µg/mL), the predominant constituents were identified as [6]-shogaol (27.43%), pellitorine (7.5%), [6]-gingerol (7.02%), and piperine (5.33%). Notably, [6]-shogaol, pellitorine, and [6]-gingerol were present in all extracts prepared with various solvents; however, piperine was exclusive to the 80% ethanol extract of TRJ 2. Consequently, piperine was designated as the chemical biomarker specific to TRJ 2 extracted with 80% ethanol (Table 2).

### 3.3. Molecular Docking Simulation Analysis

The main purpose of the docking experiment here is to use computational simulations to guide and explain the possible molecular interactions underlying the observed biological effects of the bioactive fractions mentioned above. The authors began by introducing a 5% cutoff to primarily focus on the contributions of the main components. Our hypothesis is that a metabolite detected at higher abundance is more likely to be responsible for the anti-glucosidase activity found in the traditional Thai medicine remedy. However, it comes with a significant trade-off: the exclusion of the collective effect of minor components. Still, this approach has proven beneficial for prioritizing potential bioactive molecules in GC-MS analysis, as previously reported [28]. The compounds that meet the introduced threshold are shown in Table 3. These major components were then subjected to molecular docking simulations.

**Figure 1:**
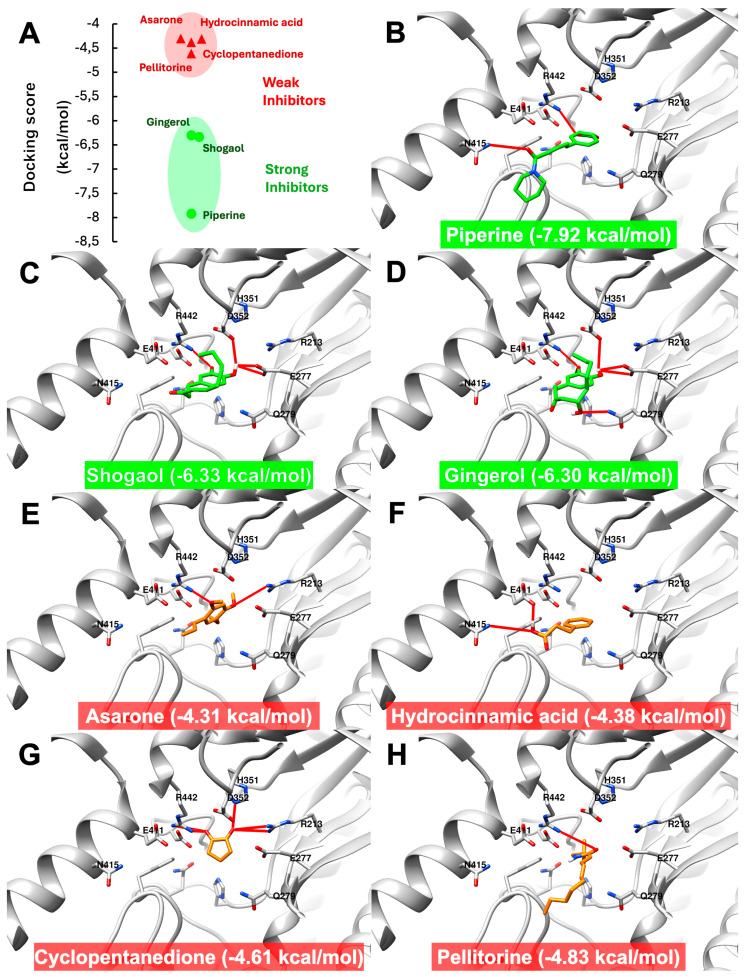
Molecular docking results of the major components found in the bioactive fractions and α-glucosidase (PDB ID: 3A4A). (**A**) Obtained docking scores of the compounds of interest. (**B**–**H**) Predicted molecular interactions of piperine, shogaol, gingerol, asarone, hydrocinnamic acid, cyclopentanedione, and pellitorine, accordingly. The red line indicates predicted hydrogen bonds between compounds of interest and the amino acids on the alpha-glucosidase enzyme.

First, the authors validated the docking protocol via a re-docking approach. The docking validation result indicated high predictive power, with an RMSD of 2.288 Å (below the acceptance criterion of 3 Å), and is provided in the Supplementary File (Figure S1). Later, the authors performed seven independent docking experiments. The docking scores obtained for each compound are shown in Figure 1. The compounds of interest were divided into two categories: predicted strong (●) and weak (▲) inhibitors, based on their docking scores (Figure 1A). The strong inhibitor group consisted of piperine, [6]-shogaol, and [6]-gingerol. Meanwhile, asarone, hydrocinnamic acid, 1,2-cyclopentanedione, and pellitorine were classified as weak inhibitors. Piperine exhibited the lowest score (−7.92 kcal/mol), indicating the most promising inhibitor among the detected major metabolites and interacting with two amino acid residues, N415 and R442 (Figure 1B and Table 4). Furthermore, [6]-shogaol and [6]-gingerol showed the same docking score level (−6.33 and −6.30 kcal/mol), indicating potent inhibition of α-glucosidase and forming five and four hydrogen bonds, respectively. Both molecules interacted with nearly identical amino acids, E277, D352, and R442, except that gingerol was able to interact with Q279 (Figure 1C,D). On the other hand, asarone, hydrocinnamic acid, 1,2-cyclopentanedione, and pellitorine showed higher scores ranging from −4.31 to −4.83 kcal/mol (Figure 1E–H), indicating a weak inhibitory effect on alpha-glucosidase. In total, there were five amino acid residues involved, such as R213, H351, E411, N415, and R442 (Table 4).

### 3.4. Density Function Theory (DFT) Analysis

**Figure 2:**
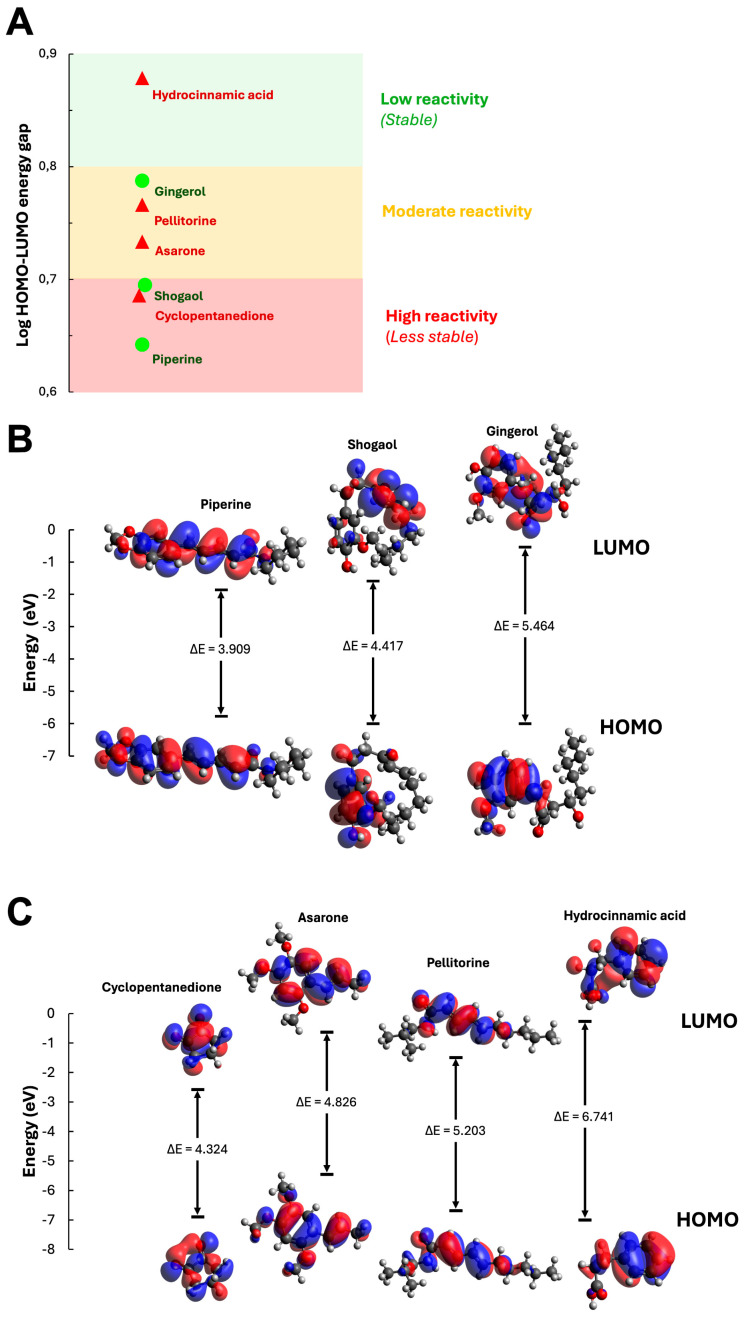
Calculated LUMO, HOMO energy levels, and HOMO-LUMO energy gaps of the seven main components, such as piperine, shogaol, gingerol, cyclopentanedione, asarone, pellitorine, and hydrocinnamic acid. (**A**) Energy landscape of the seven components HOMO–LUMO energy gap in log scale across three categories: low reactivity (green), moderate reactivity (yellow), and high reactivity (red). ● represents components with a predicted strong inhibitory effect, while ▲ represents components with a predicted weak inhibitory effect based on docking simulation. (**B**,**C**) HOMO—LUMO energy gap of three predicted strong inhibitors, such as piperine, shogaol, and ginerol, and the energy gap of four predicted weak inhibitors, such as cyclopentanedione, asarone, pellitorine, and hydrocinnamic acid, respectively. Blue represents an orbit with a positive phase, while red represents an orbit with a negative phase.

The authors aimed to use DFT parameters to evaluate compound reactivity. Four DFT parameters, such as HOMO and LUMO energy levels, the HOMO-LUMO energy gap, and molecular hardness, were evaluated as shown in Figure 2 and Table 5. The authors analyzed the DFT parameters by compound category, such as predicted strong (●) and weak inhibitor (▲) groups, as in the previous analysis. Figure 2A demonstrates the overall HOMO-LUMO energy gap landscape on a log scale, and there are three energy regions according to the obtained energy gap: low reactivity (stable), moderate, and high reactivity (less stable) regions.

Among the predicted strong inhibitor group, piperine had the smallest HOMO-LUMO energy gap (∆E = 3.909 eV) and molecular hardness (η = 1.955, Table 5), indicating the highest reactivity (i.e., less stability) within this group. Meanwhile, shogaol exhibited a moderate HOMO-LUMO energy gap (∆E = 4.909 eV, Figure 2B) and molecular hardness (η = 2.209, Table 5), indicating moderate reactivity. Finally, the gingerol showed the largest HOMO-LUMO energy gap (∆E) with 5.464 eV (Figure 2B), followed by the highest value of molecular hardness (η) of 2.732 (Table 5). This indicates that gingerol is the most stable compound (i.e., the least reactive) in the predicted strong inhibitor group.

For the predicted weak inhibitor group, cyclopentanedione exhibited the smallest HOMO–LUMO energy gap (∆E = 4.324 eV, Figure 2C) and molecular hardness (η = 2.162, Table 5), indicating the highest reactivity, less stable, within this group and the second most reactive inhibitor among the major components, only below piperine (Figure 2A,B). On the contrary, hydrocinnamic acid was a predicted weak inhibitor; the most stable inhibitor showed the largest HOMO-LUMO energy gap (∆E = 6.741 eV, Figure 2C) and molecular hardness (η = 3.371, Table 5) among all main metabolites. Asarone and pellitorin were the second- and third-compounds from the predicted weak inhibitor group, with relatively small HOMO-LUMO energy gaps (E = 4.826 and 5.203 eV, Figure 2C) and molecular hardness (η = 2.413 and 2.602; Table 5), indicating moderate reactivity similar to gingerol from the predicted strong inhibitor group (Figure 2A).

### 3.5. Linear Regression Relationship Between Anti-α-Glucosidase Activities and the Predicted TRJ 2 Derived-Inhibitors Content

**Figure 3:**
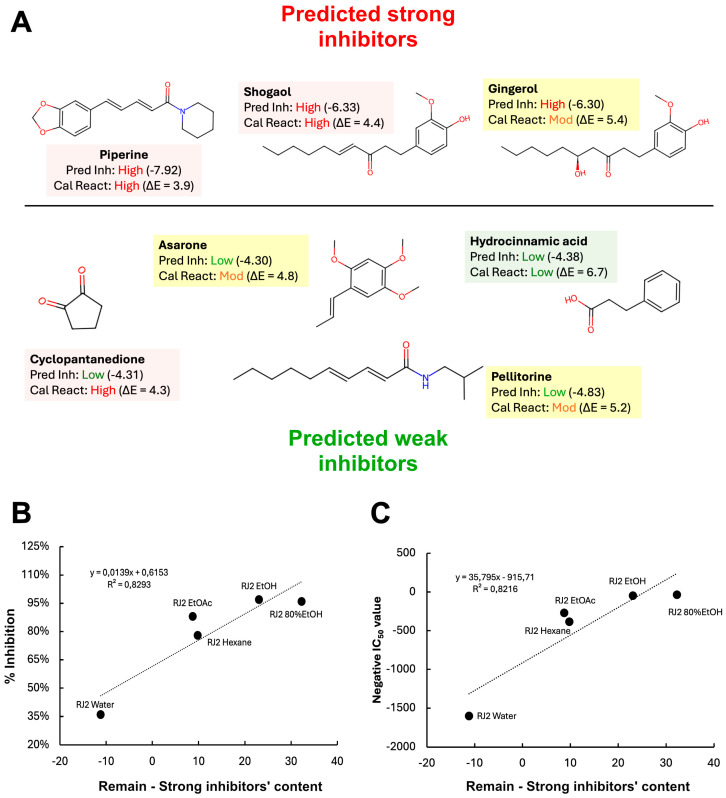
An overview of the chemical structures, predicted inhibitory effect, and predicted reactivity of major components detected in the TRJ 2 bioactive fractions, and the linear correlation between the remaining strong inhibitor content and observed %inhibition and IC_50_ value of each fraction. (**A**) Complete computational results and chemical structures of each major component, ordered by predicted inhibitory potency, while the text highlights are based on reactivity levels. Red indicates a high reactivity level, yellow indicates a moderate reactivity level, and green indicates a low reactivity level. (**B**,**C**) Linear relationship between the remaining strong inhibitor content after deducted with weak inhibitor content and the obtained anti-alpha-glucosidase activity (%inhibition and IC_50_ value).

The main objective of the computational experiments, such as molecular docking and DFT analysis, is to develop a theoretical explanation for how the major GC-MS-detected metabolites of the TRJ 2 extracts may contribute to anti-α-glucosidase activity. There were two computational layers for this theoretical explanation. The first layer was derived from molecular docking simulations, which predicted the potential inhibitory effects of the detected main components based on docking scores. The second layer was obtained from DFT analysis, which provided information on the reactivity of each major metabolite and how these inhibitors may interact with α-glucosidase when present together in a mixture. A summary of the computational results, together with the chemical structure, is provided in Figure 3A. First, the authors used docking simulation to formulate a simple hypothesis that the observed experimental inhibitory effects (% inhibition and IC_50_ values) of each fraction primarily depended on the remaining content of predicted strong inhibitors after subtracting the predicted weak-inhibitor content (as presented in Equation 1 in the Materials and Methods section). Then, the calculated residual content of the predicted strong inhibitors was plotted against the observed % inhibition and IC_50_ values from biological experiments, along with the linear regression equation (Equation (2) in the Materials and Methods section), as shown in Figure 3B,C. The high R^2^ value of around 0.82 indicated a relatively strong relationship between the predicted residual content of strong inhibitors and the observed experimental inhibitory effect, as measured by % inhibition and IC_50_ value.

However, this simple hypothesis was challenged by experimental observations from the TRJ 2 ethyl acetate and hexane extracts. Both extracts contained nearly identical amounts of predicted strong and weak inhibitors (Table 6), but the observed α-glucosidase effects differed distinctively, with % inhibition differing by 10% and IC_50_ values around 1.5 times lower for the ethyl acetate extract. To address this challenge, the authors used the second computational layer from the DFT analysis, the molecule reactivity, to explain it. Although both fractions contained similar amounts of predicted strong and weak inhibitors, hydrocinnamic acid, a predicted weak inhibitor with low calculated reactivity (stable), was present only in the TRJ 2 ethyl acetate extract. This theoretically increased the likelihood that strong inhibitors such as shogaol and gingerol would effectively block α-glucosidase activity, leading to a strong effect (higher % inhibition and lower IC_50_ value). On the other hand, the TRJ 2 hexane extract contained two predicted weak inhibitors with moderate calculated reactivity. These contributed to more competitive interactions between weak and strong α-glucosidase inhibitors, resulting in a weaker experimental effect (lower % inhibition and higher IC_50_ value).

### 3.6. TRJ 2 Complex Powder

**Figure 4:**
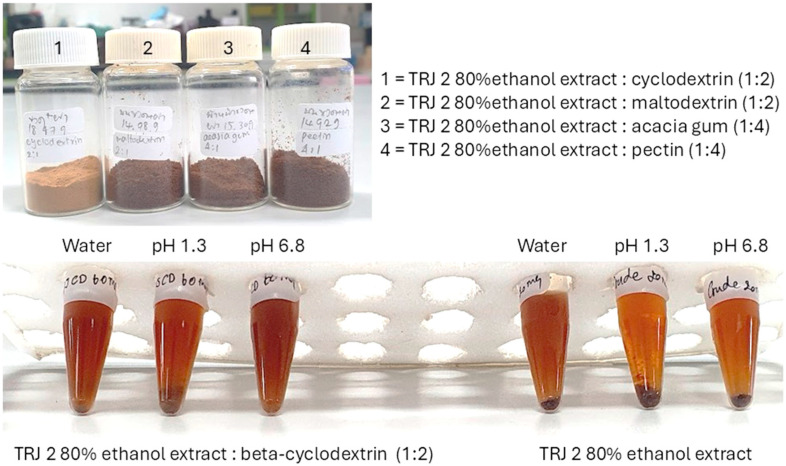
Physical appearance and solubility profiles of TRJ extract formulations. (**Top**) Power prepared with different materials: (1) β-cyclodextrin (1:2), (2) maltodextrin (1:2), (3) acacia gum (1:4), and (4) pectin (1:4). (**Bottom**) Visual comparison of solubility and color stability between the TRJ 80% ethanol extract encapsulated with β-cyclodextrin (1:2) (**left**) and the unencapsulated crude TRJ extract (**right**) dissolved in water, pH 1.3, and pH 6.8 media..

TRJ 2 was further developed into a capsule formulation. Complex compounds of the herbal powder were prepared using four binders: cyclodextrin, maltodextrin, acacia gum, and pectin. Cyclodextrin was found to be the best binder that enhanced the dissolution efficiency of the formula, especially in a pH 6.8 buffer, when prepared at a ratio of TRJ 2 extracted with 80% ethanol to cyclodextrin of 1:2 (Figure 4). The resulting complex compound yield was 4.65 g.

### 3.7. Quantification of Biomarkers in TRJ 2

**Figure 5:**
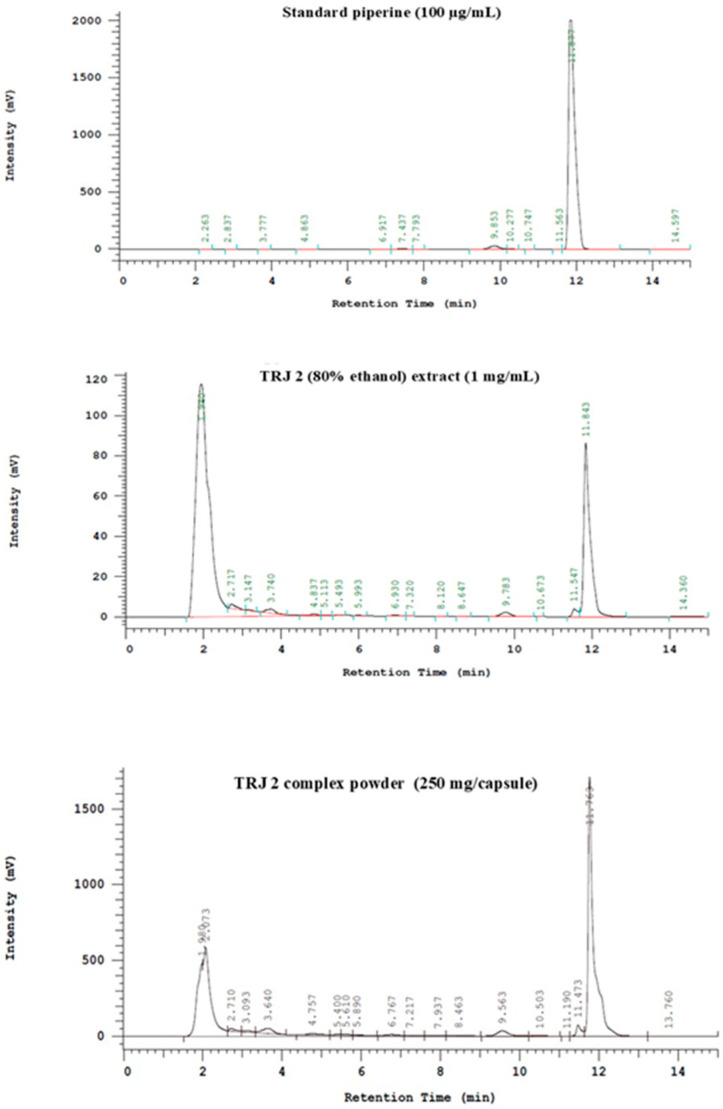
HPLC chromatograms of standard piperine (100 µg/mL), TRJ 2 80% ethanol extract (1 mg/mL), and TRJ 2 complex (250 mg/capsule). A major peak corresponding to piperine was observed at a retention time of approximately 11.5–11.8 min in both the extract and the product, consistent with the standard chromatogram, confirming the presence of piperine in the analyzed samples.

The quantification of piperine in this study was performed using HPLC technique. The TRJ 2 extracted from 80% ethanol and the powdered drug complex were examined for piperine content and compared to a piperine standard (Figure 5). The results showed that the TRJ 2 extract and the drug powder contained 3.87 mg/g and 2.53 mg/g of piperine, respectively.

### 3.8. Weight Variation Analysis

The prepared powder was encapsulated into No. 1 capsules, each containing 250 mg. An examination of the capsules revealed no visible defects and no capsule lid detachment. Weight variation analysis, conducted in accordance with the standards outlined in the THP 2012, demonstrated that among a randomly selected sample of 10 capsules, no more than two capsules exhibited a powder weight deviation exceeding 10%. The ten capsules had a mean weight of 240.6 ± 5.4 mg, and all of them were between 90 and 100% content.

### 3.9. Dissolution Analysis

**Figure 6:**
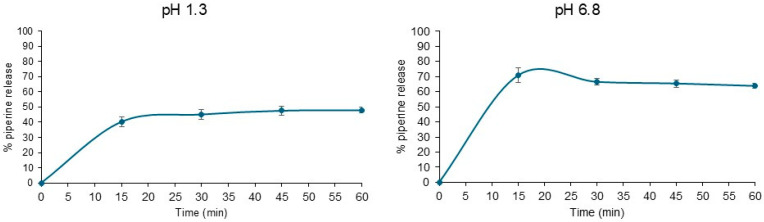
Dissolution profiles of TRJ 2 drug capsules using the USP Type-II dissolution apparatus at 100 rpm in pH 1.3 and pH 6.8. Each data point represents the average ± standard deviation (*n* = 3).

This investigation examined the solubility profile of a capsule-formulated TRJ 2 under simulated gastric (pH 1.3) and intestinal (pH 6.8) conditions at 37 °C. As depicted in Figure 6, the prepared powder complex demonstrated a higher dissolution and piperine release at pH 6.8 (70.93%) compared to pH 1.3 (40.30%) within a 15 min interval. These results correspond to the objective to improving the powder formulation’s availability and therapeutic efficacy in the intestinal environment, which will result in efficient alpha-glucosidase inhibition and a subsequent decrease in the absorption of glucose into the bloodstream.

### 3.10. Microbial Limit Test

The prepared herbal powder from the capsules tested negative for all organisms that was required following THP 2021 including Total Aerobic Microbial Count (TAMC), Total Yeasts and Molds Count (TYMC), and Bile-tolerant Gram-negative bacteria, *Escherichia coli*, *Clostridium* spp., and *Salmonella* spp. (Table 7).

### 3.11. Heavy Metal Contamination Test

This study conducted tests to detect heavy metals in the prepared herbal powder from capsules. The experimental results showed no contamination with arsenic (As) and mercury (Hg). Lead (Pb) was found in small amounts (0.03 ppm), which is below the standard limit (<10 ppm). Cadmium (Cd) was measured at 0.38 ppm, slightly exceeding the standard limit (standard < 0.30 ppm) (Table 7).

### 3.12. Stability Testing

**Figure 7:**
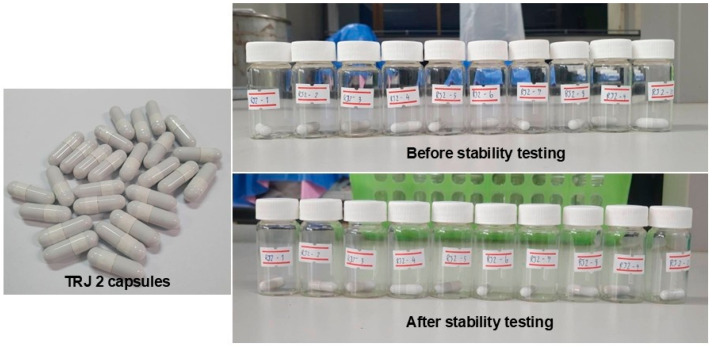
Physical appearance of TRJ 2 capsules during the stability study. TRJ 2 capsules, samples before stability testing, and samples after stability testing under the prescribed storage conditions. No noticeable changes in the external appearance of the capsules were observed after the stability study.

The capsule stability testing in this study used accelerated stability testing, a process that employs an environment with abnormally high temperatures and humidity −5 ± 2 °C (55% RH relative humidity) alternating with 45 ± 2 °C (11% RH relative humidity) to accelerate drug degradation. Physical changes such as color, odor, and capsule changes were evaluated, as well as chemical changes such as the degradation of piperine, a biomarker for the TRJ 2 herbal medicine. The experimental results showed that the capsules of the TRJ 2 did not change in color or odor (Figure 7), and the amount of piperine after accelerated testing was 2.28 mg/g, which is close to the amount of piperine before accelerated testing (2.53 mg/g).

## 4. Discussion

Traditional medicinal formulations typically contain several species of herbal constituents, based on the premise that the synergistic combination of these herbs enhances their therapeutic efficacy. This study highlights the ethnobotanical knowledge involved in the preparation of multi-species herbal remedies for diabetes management. We compiled a collection of 62 documented Thai herbal remedies and evaluated their potential anti-diabetic properties by assessing their anti-α-glucosidase enzymatic activity. The results showed that TRJ 2 (IC_50_ = 34.32 µg/mL) was more effective against the α-glucosidase enzyme than acarbose (IC_50_ = 242.94 µg/mL). Acarbose is an established α-glucosidase inhibitor, initially isolated from bacteria belonging to the genus *Actinoplanes*. α-Glucosidase inhibitors attenuate the digestion and absorption of carbohydrates by competitively inhibiting the enzymatic activity of glucosidase. As a result, postprandial peaks in blood glucose levels are diminished, thereby facilitating improved glycemic regulation [29]. The TRJ 2 formulation consists of three botanical components: *Z. officinale* (rhizome), *P. ribesioides* (climber), and *P. sarmentosum* (root), in a ratio of 1:1:1. Based on in vitro assessments, the extract of *P. ribesioides* demonstrated the most potent α-glucosidase inhibitory activity, followed by *P. sarmentosum* and *Z. officinale*.

*P. ribesioides* is extensively utilized in traditional medicinal practices in Southeast Asia. The roots of the plant have been employed to treat ailments associated with asthma, diarrhea, and abdominal pain. Additionally, the leaves are traditionally used to correct imbalances related to bodily wind (air) elements, alleviate chest congestion, and facilitate phlegm expulsion. The flowers have also been used in the treatment of urticaria [30]. The stem (climber) is used as a carminative, antiflatulent, and element tonic [31]. Although several reviews focusing on the specific therapeutic aspects of *P. ribesioides* have been published, none have comprehensively summarized its beneficial effects on T2DM and related complications. Consequently, the identification of α-glucosidase inhibitory activity in *P. ribesioides* in this study represents a promising foundation for subsequent research into natural bioactive compounds for diabetes management. In contrast to *P. ribesioides*, *P. sarmentosum* and *Z. officinale* have demonstrated multiple anti-diabetic properties. Most of the pharmacological research on *P. sarmentosum* has focused on its leaves [32]. Additionally, the entire plant, including its roots, leaves, and fruits, has been traditionally employed in the management of various ailments such as colds, gastritis, rheumatoid joint pain, abdominal discomfort, dental pain, diabetes mellitus, helminthic infections, and other conditions over several decades [33]. In this study, the root of *P. sarmentosum*, as used in TRJ 2, was selected. Its extract showed inhibitory activity against the α-glucosidase enzyme, with an IC_50_ value of 79.79 µg/mL. *Z. officinale*, commonly known as ginger, is an extensively utilized natural substance recognized for its dual role as a culinary spice and therapeutic agent. It has been traditionally employed in the management of various ailments, including nausea, dysentery, heartburn, flatulence, diarrhea, anorexia, infectious diseases, cough, and bronchitis. Additionally, ginger exhibits notable anti-inflammatory and antioxidant activities, contributing to its pharmacological efficacy [33]. Regarding its antidiabetic effects, numerous studies have been conducted. Although the inhibitory effect of ginger extract on α-glucosidase activity was modest in this study, previous investigations have demonstrated that ginger possesses significant antidiabetic properties. A daily dosage of 1.2 g of ginger administered over a three-month period led to a statistically significant decrease in fasting blood sugar (FBS) and HbA1c levels among individuals with T2DM. Similarly, another study reported notable reductions in FBS and HbA1c following 10 weeks of supplementation with 2 g of ginger per day in patients with T2DM [34,35,36].

The TRJ 2 formulation demonstrated significant inhibitory efficacy, suggesting a potential synergistic interaction among the plant constituent in suppressing α-glucosidase activity. Therefore, further investigations were conducted using polarity-based extraction methods to evaluate its α-glucosidase inhibitory capacity, analyze its chemical composition, and identify potential biomarkers in various solvent extracts. In this study, the chemical compositions were employed by GC–MS analysis, which is a fundamental and widely accessible analytical technique for the identification of volatile and semi-volatile compounds. However, GC–MS has limitations, particularly in the detection and characterization of non-volatile, thermally unstable, and highly polar phytochemicals. Consequently, important classes of secondary metabolites, including flavonoids, phenolic acids, glycosides, saponins, and certain alkaloids. Therefore, future studies should utilize other techniques, such as UHPLC–QTOF–MS/MS or UHPLC–Orbitrap–MS/MS, to obtain more accurate research results. In addition, some detected compounds may represent contaminants, analytical artifacts, or constituents not originating from the plant material. Therefore, in this study, only compounds with a Match Factor greater than 80 and those previously reported in the studied plant or related plant species in the literature were considered and presented. The extraction results showed that the TRJ 2 extract obtained with 80% ethanol exhibited the most potent inhibitory activity against α-glucosidase. This was followed by extracts obtained using ethanol, ethyl acetate, and hexane fractions, respectively. Analysis of the chemical profiles of all solvent extracts revealed that [6]-shogaol was the predominant compound in each extract, except for the TRJ 2 water extract, in which 1,2-cyclopentanedione was identified as the principal constituent. Although the four extracted fractions (80% ethanol, ethanol, ethyl acetate, and hexane) shared the same major compound, their α-glucosidase inhibitory activities differed. Several compounds identified in TRJ 2 have previously been reported to possess potential hypoglycemic or antidiabetic activities. [6]-Shogaol and [6]-Gingerol, the major bioactive constituents of ginger, have been widely recognized for their hypoglycemic potential, including enhancement of insulin sensitivity, inhibition of carbohydrate-hydrolyzing enzymes, and reduction in oxidative stress [37]. Piperine, a principal alkaloid from black pepper, has likewise demonstrated significant antidiabetic activity and was identified as a potent compound in molecular docking studies [38]. In addition, cyclopentanedione derivatives (3-methyl-1,2-cyclopentanedione) has been reported to act as a peroxisome proliferator-activated receptor gamma (PPARγ) agonist. PPARγ agonists are recognized as insulin sensitizers and are widely utilized in the management of type 2 diabetes mellitus [39]. Hydrocinnamic acid has demonstrated potential in improving glucose metabolism through its phenolic antioxidant properties [40]. Asarone has been reported to possess antidiabetic activity, potentially through the modulation of glucose metabolism pathways, particularly when tested in combination with metformin [41]. Pellitorine has also shown bioactive properties related to metabolic regulation [42].

This variation may be attributed to the presence of other compounds within each extract. To elucidate this discrepancy, molecular docking was employed to predict the anti-α-glucosidase activity of metabolites present in each extract. The potential mechanisms of action were deduced from compounds identified by GC–MS analysis. Only major constituents with more than 5% abundance were considered for the docking experiment. In reality, the excluded minor compounds may still exert synergistic effects that contribute to the overall biological activity. However, they were not included in our computation. Based on molecular docking analysis, the compounds were classified into “strong” and “weak” inhibitors according to their docking scores. Piperine was identified as the most promising inhibitor due to its lowest docking score and specific interactions with amino acid residues N415 and R442. Similarly, [6]-shogaol and [6]-gingerol demonstrated notable binding characteristics, particularly given their comparable binding affinities and interaction profiles with key amino acid residues such as E277, D352, and R442. The E277 residue functions as an acid–base catalyst, protonating the oxygen of the glycosidic bond to promote bond cleavage, whereas D352 serves as the nucleophile, attacking the anomeric carbon of the substrate to form a transient covalent bond with the glucose molecule [16]. R442 is an amino acid residue located within the catalytic domain of the α-glucosidase enzyme. It serves as a primary binding site for inhibitory molecules, frequently engaging in hydrogen- bonding interactions with compounds such as flavonoids. This interaction impedes the enzymatic hydrolysis of carbohydrates, thereby contributing to the regulation of hyperglycemia [43]. Furthermore, our computational findings in this study agree with a previous report by Temrangsee et al. (2019) [44] on the anti-α-glucosidase activity of piperine, [6]-shogaol, and [6]-gingerol, with IC_50_ values of 0.16 mM, 0.92 mM, and 1.14 mM, respectively. While in Temrangsee’s study, the IC_50_ value of 0.21 mM was reported for acarbose (the standard reference). On the other hand, compounds such as asarone, hydrocinnamic acid, cyclopentanedione, and pellitorine, were able to form hydrogen bonds with various amino acid residues but exhibited lower binding affinities than those of the strong inhibitors. Therefore, these compounds were categorized as exhibiting a potentially relatively weak inhibitory effect on α-glucosidase.

Moreover, density functional theory (DFT) parameters were employed to evaluate the chemical reactivity of the studied compounds. Key descriptors in DFT, including HOMO and LUMO energy levels, the HOMO–LUMO energy gap (ΔE), and molecular hardness (η), were analyzed. These parameters are widely associated with molecular stability and reactivity, with smaller ΔE and lower η typically indicating higher reactivity and lower stability [45]. Within the predicted strong inhibitor group, piperine exhibited the highest reactivity. This suggests that piperine may interact more readily with the target system, potentially enhancing its inhibitory performance. In contrast, [6]-shogaol and [6]-gingerol showed moderate and lower reactivity, respectively. This trend suggests that, despite being classified as strong inhibitors, [6]-shogaol and [6]-gingerol may rely on mechanisms other than intrinsic electronic reactivity. A similar trend was observed in the predicted weak inhibitor group. 1,2-Cyclopentanedione showed the highest reactivity within this group and ranked second overall after piperine. This finding suggests that some weak inhibitors may still possess considerable reactivity. Conversely, hydrocinnamic acid exhibited high stability and minimal reactivity, which is consistent with its weak inhibitory classification. Asarone and pellitorine exhibited moderate reactivity comparable to that of gingerol. These results demonstrate that, while DFT-derived reactivity parameters generally align with inhibitor classification, some overlap exists between strong and weak inhibitors. This suggests that inhibitory activity can be influenced by multiple factors, such as structural features and properties, molecular interactions, and binding affinity.

By integrating molecular docking and density functional theory (DFT) analyses, this study provides a comprehensive elucidation of the mechanisms underlying the α-glucosidase inhibitory activity of TRJ 2 extracts. The correlation between residual strong inhibitor content and inhibitory efficacy was substantial (R^2^ ≈ 0.82) across most fractions, indicating that higher residual strong inhibitor levels generally predict greater inhibitory activity. Nonetheless, notable exceptions emerged, particularly between the ethyl acetate and hexane extracts, which contained comparable amounts of predicted strong and weak inhibitors yet demonstrated markedly different inhibitory effects. The ethyl acetate extract contained hydrocinnamic acid, a predicted weak inhibitor with low reactivity, which was absent from in the hexane fraction. The presence of hydrocinnamic acid in the ethyl acetate fraction appears to mitigate competitive interactions among inhibitors, thereby facilitating the predominant action of strong inhibitors such as [6]-shogaol and [6]-gingerol, resulting in a higher percentage inhibition and a lower IC_50_ value from the ethyl acetate extracts. Conversely, the hexane extract contained weak inhibitors with moderate reactivity, capable of engaging in competitive interactions that likely attenuated overall inhibitory potency. These results underscore that inhibitory efficacy is influenced not only by the presence and quantity of inhibitors but also significantly by their reactivity profiles and interaction dynamics within the mixture. The reactivity data derived from DFT calculations provide critical insights into the observed variations in bioactivity, capturing effects that were not fully elucidated by docking scores alone. However, the calculated remaining strong inhibitor content represents a theoretical estimation based on the relative abundance of compounds reported in the literature. Furthermore, molecular docking and DFT analyses provide computational pre-dictions of potential molecular interactions and electronic properties but cannot independently confirm biological efficacy or synergistic effects. Therefore, the present study findings are regarded as supportive and hypothesis-generating, and further studies involving compound isolation, enzyme kinetics, and in vivo validation are required to establish causal relationships between phytochemical composition and biological activity. Following the identification of the 80% ethanol extract of TRJ 2 as exhibiting the most potent α-glucosidase inhibitory activity, the authors proceeded to formulate this extract into capsules for ready use. The complex constituents of the herbal powder were combined with different binders. Among these, cyclodextrin demonstrated superior enhancement of the extract’s dissolution efficiency, particularly in phosphate buffer at pH 6.8. Cyclodextrins are cyclic oligosaccharides with various derivatives that are widely utilized in pharmaceutical applications. Furthermore, cyclodextrin exhibits low toxicity due to its minimal absorption in the gastrointestinal tract, which limits systemic exposure upon oral administration. This favorable safety profile renders cyclodextrin suitable for incorporation into diverse pharmaceutical formulations, including tablets, capsules, and oral suspensions [46]. TRJ 2 capsules were formulated at a dosage of 250 mg per capsule. Upon conducting the uniformity of weight test, it was observed that 10 capsules of the TRJ 2 formulation fell within the established acceptable weight range. This finding suggests that the pre-encapsulation processes, including the preparation of the formulated powder complex and the filling of hard capsules, were executed with precision and consistency. The dissolution test evaluates the extent and rate of solution formation from a capsule and is crucial for determining drug bioavailability and therapeutic efficacy [47]. As piperine exhibits low aqueous solubility but high membrane permeability, its dissolution rate constitutes the primary rate-limiting step for absorption. In this study, significantly higher dissolution was observed at pH 6.8, where approximately 70% of piperine was released within 15 min, compared with only 1.05% released from conventional piperine capsules within the same time frame [48]. The dissolution profile of the TRJ 2 formulation demonstrated that cyclodextrin inclusion complexes could significantly enhance the dissolution of piperine in the medium, potentially leading to improve in vivo bioavailability. Moreover, the TRJ 2 powder complex demonstrated the absence of microbial contamination, aligning with established THP standards [25]. Analytical assessments of heavy metal contamination indicated that only cadmium slightly exceeded prescribed safety limits. In Thailand, existing literature reports cadmium contamination in water and soil within agricultural regions. Phytoremediation studies demonstrate that plants tend to bioaccumulate cadmium at higher concentrations compared to other heavy metals, particularly when cultivated in contaminated soils, owing to efficient uptake mechanisms across various tissues [49]. Cadmium primarily accumulates in root systems before translocation to aerial parts, with distribution influenced by species-specific physiological mechanisms. Contamination levels are most pronounced in the topsoil (approximately 10 cm depth), where root exposure is maximal, whereas deeper soil layers exhibit comparatively lower contamination. Consequently, in the context of the TRJ 2 formulation, *P. sarmentosum*, particularly its roots, may contribute to cadmium accumulation due to its role as a key ingredient [50]. Finally, the accelerated stability study was conducted using 10 representative samples stored under conditions of −5 ± 2 °C/55% RH and 45 ± 2 °C/11% RH for 12 days. No significant changes in physical characteristics were observed in any samples. Additionally, the piperine content after stability testing was measured at 2.28 mg/g, indicating minimal degradation compared to the initial concentration of 2.53 mg/g prior to testing. These results suggest that the formulation maintains its chemical integrity under accelerated storage conditions.

Despite these promising findings, several limitations should be considered. The phytochemical characterization of TRJ 2 was based primarily on GC–MS analysis, which may not fully capture non-volatile, thermally unstable, or highly polar metabolites. Therefore, the absence of complementary LC–MS/MS analyses may have limited the comprehensiveness of the metabolite profile. In addition, although the HPLC method was suitable for piperine quantification in the present study, future work should include comprehensive method validation, including assessments of specificity, accuracy, precision, linearity, robustness, and detection limits in accordance with established analytical guidelines, to further strengthen the reliability of quantitative measurements. Furthermore, the molecular docking, DFT analyses, and the proposed “remaining strong inhibitor content” model should be regarded as preliminary approaches that provide mechanistic hypotheses regarding the observed bioactivity. Future research should incorporate comprehensive metabolomic analyses, bioactivity-guided isolation of active compounds, enzyme kinetic studies, and in vivo validation to confirm the proposed mechanisms, evaluate potential synergistic interactions, and establish causal relationships between phytochemical composition and biological activity.

## 5. Conclusions

The TRJ 2 elixir is a traditional Thai polyherbal formulation composed of three plants with inhibitory effects on α-glucosidase enzymes. Among its various preparations, the 80% ethanol extract demonstrates the most potent α-glucosidase inhibitory activity. The extract is primarily composed of [6]-shogaol, [6]-gingerol, and piperine as its principal constituents. Computational investigations, including molecular docking and density functional theory analyses, have demonstrated that these compounds act synergistically to inhibit α-glucosidase enzymes, yielding a lower IC_50_ value for TRJ 2 compared to the reference drug acarbose. Furthermore, the modernization of traditional medicinal formulations into more convenient and user-friendly dosage forms is increasingly important in contemporary healthcare contexts. Accordingly, this study reformulated TRJ 2 from a traditional decoction into a capsule dosage form. The incorporation of cyclodextrin in the preparation of TRJ 2 powder enhanced the solubility of the active constituent piperine, particularly at pH 6.8, which corresponds to the intestinal environment where α-glucosidase activity is predominant. Evaluation of the physicochemical and quality attributes of the TRJ 2 capsules indicated compliance with most Thai Herbal Pharmacopoeia (THP) standards; however, a marginal exceedance in cadmium content was observed. This limitation may be reduced through careful quality control and screening of raw materials prior to capsule manufacturing to minimize the risk of contamination. Overall, these findings provide scientific validation for the significance of the traditional herbal formulation TRJ 2 and underscore its potential for development as an alternative therapeutic option for individuals with diabetes or prediabetes. Nevertheless, further investigations, particularly in vivo toxicity studies, are warranted to enhance its safety profile and strengthen consumer confidence in its future application.

## Figures and Tables

**Table 1:** Percentage yield and inhibitory effects of extracts from Thai Rejuvenating Remedy 2 (TRJ 2) on α-glucosidase activity.

Extract	Solvent Extraction	% Yield	% Inhibition (2 mg/mL)	IC_50_ (µg/mL)
*Z. officinale*	80% ethanol	13.57	51.86 ± 0.87	not detected
*P. ribesioides*	80% ethanol	6.27	97.78 ± 0.76	21.99
*P. sarmentosum*	80% ethanol	5.50	92.74 ± 0.69	79.79
TRJ 2	80% ethanol	11.59	96.67 ± 0.19	34.32
TRJ 2	Hexane	1.46	78.20 ± 0.37	384.23
TRJ 2	Ethyl acetate	1.05	88.65 ± 0.93	270.49
TRJ 2	ethanol	7.87	97.14 ± 0.42	48.88
TRJ 2	Water	2.02	36.84 ± 3.78	not detected
Piperine		not detected	not detected	0.22
Acarbose		not detected	89.81 ± 0.60	242.94

**Table 2:** Chemical composition of Thai Rejuvenating Remedy 2 (TRJ 2) extracted using different solvents.

No	RT	Compound Name	% of Total				
			TRJ 2 80%EtOH	TRJ 2 Hexane	TRJ 2 EtOAc	TRJ 2 EtOH	TRJ 2 Water
1	4.6234	1,2-Cyclopentanedione	-	-	-	-	10.78 *
2	6.071	2-Cyclopenten-1-one, 2-hydroxy-3-methyl-	-	-	-	-	1.63
3	6.5211	1,2,3-Propanetriol, 1-acetate	-	-	1.16	-	-
4	6.6219	2,5-Dimethyl-4-hydroxy-3(2H)-furanone	-	-	-	-	1.86
5	7.8591	2-Cyclopenten-1-one, 3-ethyl-2-hydroxy-	-	-	-	-	1.17
6	8.1720	2-Acetyl-2-hydroxy-.gamma.-butyrolactone	-	-	-	-	1.82
7	8.4100	4H-Pyran-4-one, 2,3-dihydro-3,5-dihydroxy-6-methyl-	-	-	-	1.09	4.89
8	9.6846	Catechol	-	-	-	-	3.82
9	10.2765	2-Hydroxypropane-1,3-diyl diacetate	-	-	-	1.69	-
10	11.1719	1,2-Benzenediol, 3-methyl-	-	-	-	-	3.15
11	12.9010	Hydrocinnamic acid	-	1.58	13.05	5.03	5.48
12	13.3719	Phenol, 2,6-dimethoxy-	-	-	-	-	1.45
13	16.5728	Benzene, 1-(1,5-dimethyl-4-hexenyl)-4-methyl-	-	2.9	-	-	-
14	17.2016	.beta.-Bisabolene	-	1.12	-	-	-
15	17.5639	Cyclohexene, 3-(1,5-dimethyl-4-hexenyl)-6-methylene-, [S-(R *,S *)]-	-	1.48	-	-	-
16	18.2511	Ethyl. alpha.-d-glucopyranoside	2.58	-	-	3.58	-
17	18.8393	2-Butanone, 4-(4-hydroxy-3-methoxyphenyl)-	1.14	-	-	-	-
18	18.9724	caryophyllene oxide	-	1.12	-	-	-
19	19.5922	.beta.-Asarone	1.79	-	-	-	-
20	20.3394	Butan-2-one, 4-(3-hydroxy-2-methoxyphenyl)-	-	2.43	1.91	-	2.29
21	21.1113	Asarone	-	5.22	3.09	-	-
22	21.2657	Khusinol	-	-	-	1.75	-
23	22.2333	Acorenone B	1.32	-	-	-	-
24	22.2977	Coniferyl alcohol,Z-	-	-	-	1.08	-
25	24.7743	2,4-Decadienamide, N-isobutyl-, (E,E)-(Pellitorine (6CI))	7.5	6.03	2.8	1.36	-
26	26.8873	n-Hexadecanoic acid	-	1.01	2.55	3.79	-
27	25.9448	Hexadecanoic acid, ethyl ester	2.59	-	-	1.37	-
28	28.1329	(2E,4E)-1-(Pyrrolidin-1-yl)deca-2,4-dien-1-one	1.14	2.25	1.69	-	-
29	28.5329	(2E,4E)-1-(Piperidin-1-yl)deca-2,4-dien-1-one	1.31	-	-	-	-
30	28.9623	Linoleic acid ethyl ester	2.39	-	-	-	-
31	29.0741	Ethyl Oleate	2.11	-	-	-	-
32	29.9419	(E)-1-(4-Hydroxy-3-methoxyphenyl)dec-3-en-5-one ([6]-Isoshogaol)	3.03	1.89	1.71	2.6	1.67
33	30.0440	9,12-Octadecadienoic acid (Z,Z)-	-	-	-	1.07	-
34	30.1548	Oleic Acid	-	-	1.37	2.26	-
35	31.1210	1-(4-Hydroxy-3-methoxyphenyl)dec-4-en-3-one ([6]-Shogaol)	27.43 *	10.87 *	11.16 *	18.43 *	5.08
36	32.6503	5-Hydroxy-1-(4-hydroxy-3-methoxyphenyl)decan-3-one ([6]-Gingerol)	7.02	10.16	10.6	9.66	1.79
37	34.1385	1-(4-Hydroxy-3-methoxyphenyl)-3,5-decanediol	1.03		1.83	1.54	-
38	34.4502	1-(4-Hydroxy-3-methoxyphenyl)dodec-4-en-3-one	3.31	2.54	1.84	2.58	-
39	34.8385	(3R,5S)-1-(4-Hydroxy-3-methoxyphenyl)decane-3,5-diyl diacetate	1.53	1.95	-	1.01	-
40	36.2031	(E)-5-(Benzo [d] [1,3] dioxol-5-yl)-1-(piperidin-1-yl)pent-2-en-1-one	1.46	-	-	-	-
41	36.2248	Hexadecanoic acid, 2-hydroxy-1-(hydroxymethyl)ethyl ester	-	-	-	1.49	-
42	37.5795	1-(4-Hydroxy-3-methoxyphenyl)tetradec-4-en-3-one	3.1	3.97	2.49	3.28	-
43	37.6373	5-Hydroxy-1-(4-hydroxy-3-methoxyphenyl)dodecan-3-one	-	1.21	-	1.09	-
44	39.8300	(E)-3,7-Dimethylocta-2,6-dien-1-yl palmitate	-	1.66	-	-	-
45	40.0441	Piperine	5.33	-	-	-	-
46	42.1820	(2E,4E,8E)-9-(Benzo [d] [1,3] dioxol-5-yl)-1-(piperidin-1-yl)nona-2,4,8-trien-1-one	-	-	2.23	-	-
47	42.2177	(9Z,12Z)-(E)-3,7-Dimethylocta-2,6-dien-1-yl octadeca-9,12-dienoate	-	3.69	-	-	-
48	47.3646	Stigmasterol	-	2.44	1.29	-	-

* major compound; EtOH: ethanol; EtOAc: ethyl acetate.

**Table 3:** Major components in the bioactive fractions of the TRJ 2 formulation with more than 5% content.

Compound Name	% of Total				
	TRJ 2 80%EtOH	TRJ 2 EtOH	TRJ 2 EtOAc	TRJ 2 Hexane	TRJ 2 Water
1,2-Cyclopentanedione	-	-	-	-	10.78
Hydrocinnamic acid	-	5.03	13.05	-	5.48
Asarone	-	-	-	5.22	-
Pellitorine	7.5	-	-	6.03	-
[6]-Shogaol	27.43	18.43	11.16	10.87	5.08
[6]-Gingerol	7.02	9.66	10.60	10.16	-
Piperine	5.33	-	-	-	-

**Table 4:** Summary of molecular docking result, including docking score, H-bond, and relevant amino acid residues on the alpha-glucosidase enzyme.

Compounds	Docking Score [kcal/mol]	H-Bonds	Amino Acid Residues
Predicted Strong inhibitors			
Piperine	−7.92	2	N415 (1), and R442 (1)
[6]-Shogaol	−6.33	4	E277 (2), D352 (1) and R442 (1)
[6]-Gingerol	−6.30	5	E277 (2), Q279 (1), D352 (1), and R442 (1)
Predicted Weak inhibitors			
Pellitorine	−4.82	1	R442 (1)
1,2-Cyclopentanedione	−4.61	5	R213 (2), H351 (1), and R442 (2)
Hydrocinnamic acid	−4.38	2	E411 (1) and N415 (1)
Asarone	−4.31	2	R213 (1) and R442 (1)

() indicates the number of predicted hydrogen bonds of the mentioned amino acid residue by the docking simulation.

**Table 5:** Calculated DFT parameters (LUMO and HOMO energy levels, HOMO-LUMO energy gap, and molecular hardness) of predicted strong inhibitors and predicted weak inhibitors.

Compounds	LUMO	HOMO	Energy Gap (ΔE)	Hardness (η)
Predicted Strong inhibitors				
Piperine	−1.861	−5.770	3.909	1.955
[6]-Shogaol	−1.584	−6.001	4.417	2.209
[6]-Gingerol	−0.534	−5.998	5.464	2.732
Predicted Weak inhibitors				
1,2-Cyclopentadione	−2.577	−6.901	4.324	2.162
Asarone	−0.629	−5.455	4.826	2.413
Pellitorine	−1.487	−6.690	5.203	2.602
Hydrocinnamic acid	−0.261	−7.002	6.741	3.371

**Table 6:** Summary of the remaining strong inhibitor content after deducting the weak inhibitor content of each fraction and its inhibitory effects, including % inhibition and IC_50_ values.

Compound Name	% of Total				
	TRJ 2 80%EtOH	TRJ 2 EtOH	TRJ 2 EtOAc	TRJ 2 Hexane	TRJ 2 Water
Predicted weak inhibitor					
1,2-Cyclopentanedione	-	-	-	-	10.78
Hydrocinnamic acid	-	5.03	13.05	-	5.48
Asarone	-	-	-	5.22	-
Pellitorine	7.5	-	-	6.03	-
Predicted strong inhibitor					
[6]-Shogaol	27.43	18.43	11.16	10.87	5.08
[6]-Gingerol	7.02	9.66	10.6	10.16	-
Piperine	5.33	-	-	-	-
Strong inhibitor content	39.78	28.09	21.76	21.03	5.08
Weak inhibitor content	7.5	5.03	13.05	11.25	16.26
Remain—Strong inhibitor content	32.28	23.06	8.71	9.78	−11.18
Inhibitory Effect					
% inhibition	96.00%	97.00%	88.00%	78.00%	36.00%
IC_50_	34	48	270	384	n.d. (999 *)

* indicates the IC_50_ value of 999 was used instead of n.d. (not detected) in the linear regression analysis for the TRJ 2 extracts since its IC_50_ value was not detected due to low % inhibition.

**Table 7:** Microbial and heavy metal contamination results in TRJ 2 prepared herbal powder from capsules.

Organism	Limit Test (THP)	Total Amount
Total aerobic microbial count	<5 × 10^5^ CFU/g	Not found
Total yeast and molds count	<5 × 10^5^ CFU/g	Not found
Bile-tolerant Gram-negative bacteria	<10^3^ CFU/g	Not found
*Escherichia coli*	Not found	Not found
*Salmonella* spp.	Not found/10 g	Not found
*Clostridium* spp.	Not found/1 g	Not found
**Heavy metal**		
Arsenic (As)	<4 ppm	Not found
Lead (Pb)	<10 ppm	0.03 ppm
Cadmium (Cd)	<0.3 ppm	0.38 ppm
Mercury (Hg)	<0.5 ppm	Not found

## Data Availability

The original contributions presented in this study are included in the article and Supplementary Material. Further inquiries can be directed to the corresponding author.

## Supplementary Materials

The following supporting information can be downloaded at: https://www.mdpi.com/article/10.3390/life16071084/s1, Figure S1: Molecular docking validation outcome from the established docking protocol used in this study. The RMSD value of the redocked ligand, in black, is 2.288 Å compared to the ligand’s original pose (co-crystal structure position), presented in green. The accepted criterion for redocking validation is a distance of less than 3 Å.

## Abbreviations

The following abbreviations are used in this manuscript:

TRJ	Thai Rejuvenation Remedies
DFT	Density Functional Theory
THP	Thai Herbal Pharmacopoeia (THP) Standards

## References

[1] Bruins M.J., Van Dael P., Eggersdorfer M. (2019). The role of nutrients in reducing the risk for noncommunicable diseases during aging. Nutrients.

[2] Vichitkunakorn P., Bunyanukul W., Apiwan K., Tanasanchonnakul D., Sittisombut M. (2025). Prevalence of non-communicable disease risk factors and their association with economic status: Findings from the 2021 health behaviour of population survey in Thailand. Glob. Health Action.

[3] Veit M., van Asten R., Olie A., Prinz P. (2022). The role of dietary sugars, overweight, and obesity in type 2 diabetes mellitus: A narrative review. Eur. J. Clin. Nutr..

[4] Suryasa I.W., Rodríguez-Gámez M., Koldoris T. (2021). Health and treatment of diabetes mellitus. Int. J. Health Sci..

[5] Schwartz S.S., Epstein S., Corkey B.E., Grant S.F., Gavin J.R., Aguilar R.B. (2016). The time is right for a new classification system for diabetes: Rationale and implications of the β-cell-centric classification schema. Diabetes Care.

[6] Crawford A.L., Laiteerapong N. (2024). Type 2 diabetes. Ann. Intern. Med..

[7] Teimouri A., Ebrahimpour Z., Feizi A., Iraj B., Saffari E., Akbari M., Karimifar M. (2025). Pre-diabetes and cardiovascular risk factors in NAFLD patients: A retrospective comparative analysis. Front. Endocrinol..

[8] Esquivel Zuniga R., DeBoer M.D. (2021). Prediabetes in adolescents: Prevalence, management and diabetes prevention strategies. Diabetes Metab. Syndr. Obes..

[9] Salehi B., Ata A., Anil Kumar N.V., Sharopov F., Ramírez-Alarcón K., Ruiz-Ortega A., Abdulmajid Ayatollahi S., Tsouh Fokou P.V., Kobarfard F., Amiruddin Zakaria Z. (2019). Antidiabetic potential of medicinal plants and their active components. Biomolecules.

[10] Choudhury H., Pandey M., Hua C.K., Mun C.S., Jing J.K., Kong L., Ern L.Y., Ashraf N.A., Kit S.W., Yee T.S. (2018). An update on natural compounds in the remedy of diabetes mellitus: A systematic review. J. Tradit. Complement. Med..

[11] Prabhakar P.K., Kumar A., Doble M. (2014). Combination therapy: A new strategy to manage diabetes and its complications. Phytomedicine.

[12] Dej-adisai S., Pitakbut P. (2015). Determination of α-glucosidase inhibitory activity from selected Fabaceae plants. Pak. J. Pharm. Sci..

[13] Sangkanu S., Pitakbut T., Phoopha S., Khanansuk J., Chandarajoti K., Dej-adisai S. (2024). A Comparative study of chemical profiling and bioactivities between Thai and foreign hemp seed species (*Cannabis sativa* L.) plus an in-silico investigation. Foods.

[14] Phoopha S., Wattanapiromsakul C., Pitakbut T., Dej-adisai S. (2020). Chemical constituents of *Litsea elliptica* and their alpha-glucosidase inhibition with molecular docking. Pharmacogn. Mag..

[15] Eberhardt J., Santos-Martins D., Tillack A.F., Forli S. (2021). AutoDock Vina 1.2.0: New docking methods, expanded force field, and python bindings. J. Chem. Inf. Model..

[16] Yamamoto K., Miyake H., Kusunoki M., Osaki S. (2010). Crystal structures of isomaltase from *Saccharomyces cerevisiae* and in complex with its competitive inhibitor maltose. FEBS J..

[17] Pettersen E.F., Goddard T.D., Huang C.C., Couch G.S., Greenblatt D.M., Meng E.C., Ferrin T.E. (2004). UCSF Chimera—A visualization system for exploratory research and analysis. J. Comput. Chem..

[18] Morris G.M., Huey R., Lindstrom W., Sanner M.F., Belew R.K., Goodsell D.S., Olson A.J. (2009). AutoDock4 and AutoDockTools4: Automated docking with selective receptor flexibility. J. Comput. Chem..

[19] O’Boyle N.M., Banck M., James C.A., Morley C., Vandermeersch T., Hutchison G.R. (2011). An open chemical toolbox. J. Cheminform..

[20] Neese F., Wennmohs F., Becker U., Riplinger C. (2020). The ORCA quantum chemistry program package. J. Chem. Phys..

[21] Alessa A.H. (2025). Analyzing the energetics of the four aromatic ring interactions: Theoretical study. J. Phys. Chem. A.

[22] Campisi D., Lamberts T., Dzade N.Y., Martinazzo R., Ten Kate I.L., Tielens A.G.G.M. (2021). Interaction of aromatic molecules with forsterite: Accuracy of the periodic DFT-D4 method. J. Phys. Chem. A.

[23] Hanwell M.D., Curtis D.E., Lonie D.C., Vandermeersch T., Zurek E., Hutchison G.R. (2012). Avogadro: An advanced semantic chemical editor, visualization, and analysis platform. J. Cheminform..

[24] Pitakbut T., Kayser O. (2025). Anti-infective screening of selected nine cannabinoids against *Clostridium perfringens* and Influenza A (H5N1) neuraminidases, and SARS-CoV-2 main protease and spike protein interactions. Curr. Issues Mol. Biol..

[25] Bureau of Drug and Narcotic, Department of Medical Sciences, Ministry of Public Health (2021). Thai Herbal Pharmacopoeia 2021.

[26] To N., Phuong A., Nguyen T., Pham H., Truong Q.K. (2025). Optimization of a procedure for the determination of some heavy metals in herbal medicines by atomic absorption spectroscopy. Nat. Prod. Commun..

[27] Al-Ali M., Selvakannan P.R., Parthasarathy R. (2018). Influences of novel microwave drying on dissolution of new formulated naproxen sodium. RSC Adv..

[28] Sangkanu S., Pitakbut T., Phoopha S., Khanansuk J., Chandarajoti K., Dej-adisai S. (2025). Insights into Thai and foreign hemp seed oil and extracts’ GC/MS data re-analysis through learning algorithms and anti-aging properties. Foods.

[29] Chen K. (2025). A review on metformin and acarbose as anti-diabetic drugs with representative mechanisms. MedScien.

[30] Sudmoon R. (2012). Ethnobotany and species-specific molecular markers of some medicinal sakhan (*Piper, Piperaceae*). J. Med. Plants Res..

[31] Pichiensunthon C., Jeerawongs V.C. (2004). Traditional Pharmacy Handbook.

[32] Adib A.M., Salmin N.N., Kasim N., Ling S.K., Cordell G.A., Ismail N.H. (2024). The metabolites of *Piper sarmentosum* and their biological properties: A recent update. Phytochem. Rev..

[33] Sun X., Chen W., Dai W., Xin H., Rahmand K., Wang Y., Zhang J., Zhang S., Xu L., Han T. (2020). *Piper sarmentosum* Roxb.: A review on its botany, traditional uses, phytochemistry, and pharmacological activities. J. Ethnopharmacol..

[34] Zhukovets T., Özcan M.M. (2020). A review: Composition, use and bioactive properties of ginger (*Zingiber officinale* L.) rhizoms. J. Agroaliment. Proc. Technol..

[35] Carvalho G.C.N., Lira-Neto J.C.G., Araújo M.F.M., Freitas R.W.J.F., Zanetti M.L., Damasceno M.M.C. (2020). Effectiveness of ginger in reducing metabolic levels in people with diabetes: A randomized clinical trial. Rev. Lat. Am. Enferm..

[36] Makhdoomi Arzati M., Mohammadzadeh Honarvar N., Saedisomeolia A., Anvari S., Effatpanah M., Makhdoomi Arzati R., Yekaninejad M.S., Hashemi R., Djalali M. (2017). The effects of ginger on fasting blood sugar, hemoglobin A1c, and lipid profiles in patients with type 2 diabetes. Int. J. Endocrinol. Metab..

[37] Abdul Rani A.N., Gaurav A., Lee V.S., Mad Nasir N., Md Zain S., Patil V.M., Lee M.T. (2025). Insights into biological activities profile of gingerols and shogaols for potential pharmacological applications. Arch. Pharm. Res..

[38] Baky M.H., Maamoun A.A., Nicolescu A., Mocan A., Farag M.A. (2025). Multi-targeted MS-based metabolomics fingerprinting of black and white pepper coupled with molecular networking in relation to their in vitro antioxidant and antidiabetic effects. RSC Adv..

[39] Sena S., Rasmussen I.R., Wende A.R., McQueen A.P., Theobald H.A., Wilde N., Pereira R.O., Litwin S.E., Berger J.P., Abel E.D. (2007). Cardiac hypertrophy caused by peroxisome proliferator- activated receptor-gamma agonist treatment occurs independently of changes in myocardial insulin signaling. Endocrinology.

[40] Muller C.J., Joubert E., Pheiffer C., Ghoor S., Sanderson M., Chellan N., Fey S.J., Louw J. (2013). Z-2-(β-d-glucopyranosyloxy)-3-phenylpropenoic acid, an α-hydroxy acid from rooibos (*Aspalathus linearis*) with hypoglycemic activity. Mol. Nutr. Food Res..

[41] Das B.K., Knott R.M., Gadad P.C. (2021). Metformin and asarone inhibit HepG2 cell proliferation in a high glucose environment by regulating AMPK and Akt signaling pathway. Futur. J. Pharm. Sci..

[42] Rohm B., Riedel A., Ley J.P., Widder S., Krammer G.E., Somoza V. (2015). Capsaicin, nonivamide and trans-pellitorine decrease free fatty acid uptake without TRPV1 activation and increase acetyl-coenzyme A synthetase activity in Caco-2 cells. Food Funct..

[43] Sanachai K., Chamni S., Nutho B., Khammuang S., Ratha J., Choowongkomon K., Puthongking P. (2025). Mechanistic study of α-mangostin derivatives as potent α-glucosidase inhibitors. Mol. Divers..

[44] Temrangsee P., Itharat A., Sattaponpan C., Pipatrattanaseree W. (2019). Inhitory effect on alpha-glucosidase activity of Benjakul, Soros Benjakul and their plant components. TMJ.

[45] Guan H., Sun H., Zhao X. (2025). Application of density functional theory to molecular engineering of pharmaceutical formulations. Int. J. Mol. Sci..

[46] Musuc A.M. (2024). Cyclodextrins: Advances in chemistry, toxicology, and multifaceted applications. Molecules.

[47] Kumadoh D., Ofori-Kwakye K. (2017). Dosage forms of herbal medicinal products and their stability considerations-an overview. J. Crit. Rev..

[48] Shao B., Cui C., Ji H., Tang J., Wang Z., Liu H., Qin M., Li X., Wu L. (2015). Enhanced oral bioavailability of piperine by self-emulsifying drug delivery systems: In vitro, in vivo and in situ intestinal permeability studies. Drug Deliv..

[49] Khamlerd C., Tengjaroenkul B., Neeratanaphan L. (2019). Abnormal chromosome assessment of snakehead fish (*Channa striata*) affected by heavy metals from a reservoir near an industrial factory. Int. J. Environ. Stud..

[50] Bouida L., Rafatullah M., Kerrouche A., Qutob M., Alosaimi A.M., Alorfi H.S., Hussein M.A. (2022). A review on cadmium and lead contamination: Sources, fate, mechanism, health effects and remediation methods. Water.

