# Supplementary material for: Therapeutic Innovation from Plant-Derived Thai Herbal Extracts: α-Glucosidase Inhibitory Activity, Mechanistic Insights and Formulation Potential of the Selected Thai Rejuvenation Remedy
Source: Life (Basel). 2026 Jun 28;16(7):1084. doi: 10.3390/life16071084 (PMC13413023; doi:10.3390/life16071084)
Supplement: Supplementary file 1 [file life-16-01084-s001.zip › life-4326150-supplementary.pdf]

<u>Supplementary file</u>

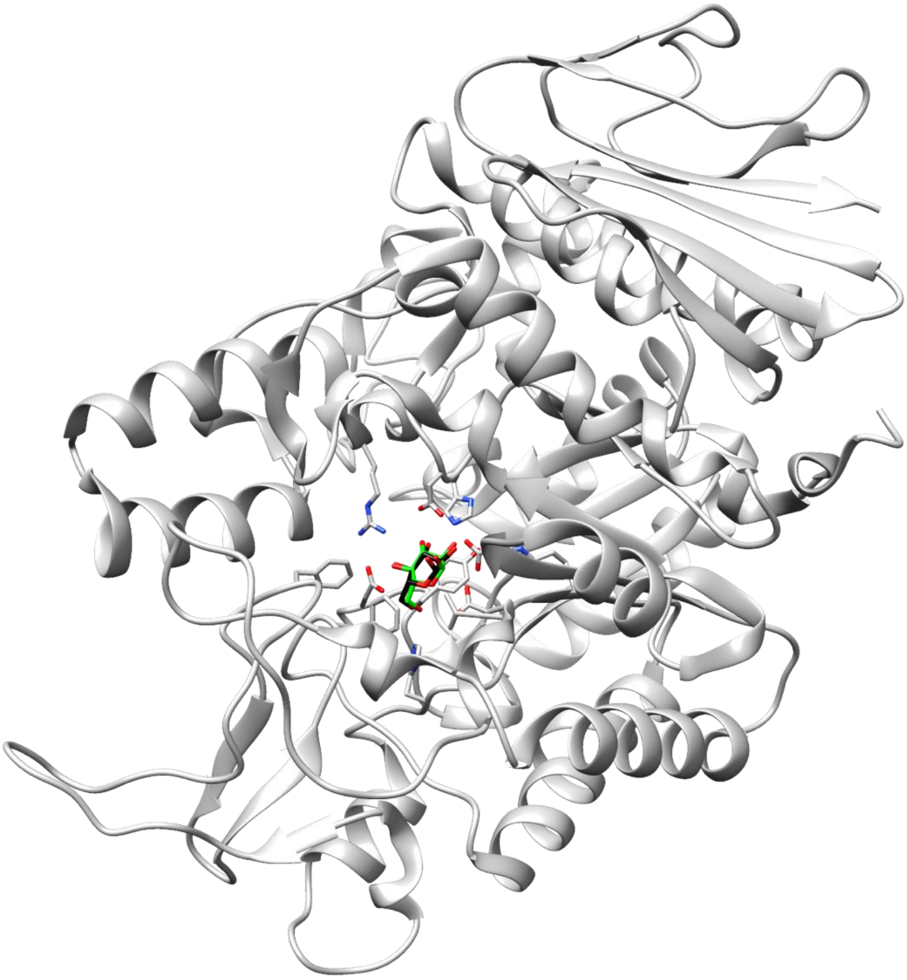

Figure S1. Molecular docking validation outcome from the established docking protocol used in this study. The RMSD value of the redocked ligand, in black, is 2.288 Å compared to the ligand's original pose (co-crystal structure position), presented in green. The accepted criterion for redocking validation is a distance of less than 3 Å.
